# Supplementary material for: Forecasting daily new infections, deaths and recovery cases due to COVID-19 in Pakistan by using Bayesian Dynamic Linear Models
Source: PLoS One. 2021 Jun 17;16(6):e0253367. doi: 10.1371/journal.pone.0253367 (PMC8211153; doi:10.1371/journal.pone.0253367)
Supplement: Attachment — Submitted filename: Responses to Reviewers.docx [file pone.0253367.s002.docx]

Authors’ Responses to Reviewers Comments about manuscript titled “FORECASTING DAILY NEW INFECTIONS, DEATHS AND RECOVERY CASES DUE TO COVID-19 IN PAKISTAN BY USING BAYESIAN DYNAMIC LINEAR MODELS”

The authors are extremely grateful to the editor and reviewers for their insightful comments/suggestions on our manuscript which are helpful to improve the quality of the manuscript. The authors’ responses are given below against each comments of reviewer 1 and 2 and 3.

|  | **Reviewers’ Comments** | **Authors’ responses** |
| --- | --- | --- |
| **S. No** | **Reviewer # 1** | |
| 1 | the main contribution and originality should be explained in more detail | There are mainly three things that were addressed in the manuscript and are the main contributions:   1. State space models have not been used for modelling and forecasting daily new infections, deaths and new recover cases due to COVID-19 in Pakistan. The plus points of state space models (especially dynamic linear models) are mentioned in the introduction of the revised manuscript. 2. More observation about the three variables have been used in this research work. This may be helpful to provide stable and robust results which can further improve the quality of forecasting. 3. Before this study, other studies provide 5, 10 days ahead forecast in Pakistan. However, this study provides 20 days ahead forecast which is comparative suitable time for planning and policies implementation. 4. In addition, this study will encourage researchers to use Bayesian Dynamic Linear Model (BDLM) for modelling and forecasting time series data about COVID-19. |
| 2 | the motivation of the approach with Bayesian Models needs further clarification, why not other models, like NNs? | In revised manuscript we add the details and motivation about BDLM in the introduction section. In this study, first time we implement BDLM for modelling and forecasting COVID-19 in Pakistan. Therefore, this study can help concerned stakeholders to make their policies according to the forecast results. However, we are planning to have a study in which we will compare different popular models (including DLM, ARIMA NNs etc.) by using COVID-19 data for Pakistan to choose the best model. |
| 3 | discussion of related work in COVID-19 should be expanded with more recent work | More papers are referred and discussed in the introduction and discussion of the revised manuscript. Emphasize is given to the most recent work about COVID-19. A list of the newly added reference is given in the end of this document. |
| 4 | Minor grammar and syntax issues need correction | The revised manuscript has been checked for grammar and syntax issues thoroughly. We hope that Quality of revised manuscript has been improved. |
| 5 | more simulation results and formal comparison of results are needed | More simulation has been drawn and the results were compared. In below table the results of 10 simulations are presented. However, in the revised manuscript, the average results of the 100 simulations are given. |
| Sim. No. \| Variable \| Min. \| Q1 \| Mean \| Median \| Q3 \| Max.; Sim01 \| Daily New Infections \| 2479 \| 3045 \| 3228 \| 3282 \| 3521 \| 4030; \| Daily Deaths \| 40 \| 49 \| 53 \| 55 \| 60 \| 71; \| Daily Recover Cases \| 281 \| 1194 \| 1794 \| 1778 \| 2196 \| 3757; Sim02 \| Daily New Infections \| 2869 \| 3222 \| 3363 \| 3364 \| 3630 \| 3716; \| Daily Deaths \| 36 \| 43 \| 52 \| 52 \| 61 \| 80; \| Daily Recover Cases \| 541 \| 1358 \| 2059 \| 1894 \| 2393 \| 3005; Sim03 \| Daily New Infections \| 2787 \| 3208 \| 3268 \| 3361 \| 3493 \| 3967; \| Daily Deaths \| 39 \| 45 \| 51 \| 52 \| 56 \| 72; \| Daily Recover Cases \| 8 \| 1194 \| 1763 \| 1666 \| 2126 \| 2930; Sim04 \| Daily New Infections \| 2498 \| 3190 \| 3368 \| 3388 \| 3632 \| 3884; \| Daily Deaths \| 37 \| 45 \| 49 \| 51 \| 59 \| 71; \| Daily Recover Cases \| 543 \| 1346 \| 1620 \| 1673 \| 2127 \| 2615; Sim05 \| Daily New Infections \| 2716 \| 3331 \| 3474 \| 3421 \| 3523 \| 4110; \| Daily Deaths \| 38 \| 43 \| 48 \| 50 \| 58 \| 72; \| Daily Recover Cases \| 561 \| 1508 \| 1791 \| 1834 \| 2169 \| 2812; Sim06 \| Daily New Infections \| 2614 \| 3093 \| 3313 \| 3357 \| 3609 \| 4095; \| Daily Deaths \| 36 \| 44 \| 50 \| 52 \| 57 \| 72; \| Daily Recover Cases \| 633 \| 1407 \| 1819 \| 1809 \| 2208 \| 3244; Sim07 \| Daily New Infections \| 2767 \| 3180 \| 3327 \| 3375 \| 3580 \| 3034; \| Daily Deaths \| 37 \| 46 \| 52 \| 52 \| 56 \| 67; \| Daily Recover Cases \| 412 \| 1323 \| 1829 \| 1818 \| 2214 \| 3047; Sim08 \| Daily New Infections \| 2343 \| 3315 \| 3473 \| 3455 \| 3713 \| 3886; \| Daily Deaths \| 36 \| 43 \| 53 \| 52 \| 61 \| 67; \| Daily Recover Cases \| 979 \| 1477 \| 1793 \| 1847 \| 2062 \| 3297; Sim09 \| Daily New Infections \| 2377 \| 3122 \| 3320 \| 3277 \| 3503 \| 4016; \| Daily Deaths \| 29 \| 44 \| 52 \| 51 \| 57 \| 70; \| Daily Recover Cases \| 1053 \| 1318 \| 1760 \| 1798 \| 1984 \| 2906; Sim10 \| Daily New Infections \| 2734 \| 3229 \| 3403 \| 3391 \| 3558 \| 3882; \| Daily Deaths \| 33 \| 48 \| 50 \| 52 \| 61 \| 66; \| Daily Recover Cases \| 861 \| 1361 \| 1768 \| 1833 \| 2105 \| 3221 *Note: Sim stands for simulation, Min., Max., Q1, Q3, stand for minimum, maximum values, first quartile and third quartile, respectively.* | | |
| 6 | the conclusions should be extended with more future work | Recommendations are provided in the conclusion of the revised manuscript. |
| 7 | More references to COVID-19 papers should be included, like: Multiple ensemble neural network models with fuzzy response aggregation for predicting COVID-19 time series: the case of Mexico. Healthcare 2020;8:181. Modeling COVID-19 epidemic in Heilongjiang province, China, Chaos Solitons Fractals, 138, 1–5. Modeling and forecasting of epidemic spreading: the case of Covid-19 and beyond. Chaos Solitons Fractals 2020;135:109794. Forecasting of COVID-19 time series for countries in the world based on a hybrid approach combining the fractal dimension and fuzzy logic. Chaos, Solitons and Fractals 140 (2020) 110242 | More papers recently published are cited in the revised manuscript. The list of the references in the revised manuscript are mentioned at the end of this document. |
|  | **Reviewer # 2** | |
| 1 | By the time authors receive this review, they will have obtained real data for the predicted values of new cases, deaths, and recovered cases. It is suggested to validate the predicted values with the real values. | Yes, we have the data now available. We made a comparison between observed and simulated data based on their average values. The simulated average values are different from their observed counterparts. However, 90 percent prediction intervals cover the observed values except daily new infection. Data/Variables \| Average New Infection \| Average Deaths \| Average Recover Cases; Observed data \| 4385 \| 71 \| 2305; Simulated data \| 3424 \| 54 \| 1879; 90% Lower Limit \| 2494 \| 36 \| 752; 90% Upper Limit \| 4351 \| 71 \| 3005 The above table show a comparison of observed and model’s simulated daily new infections, death and recover cases due to COVID-19 based on the average values. 95% prediction interval is also provided for model’s simulated data sets. We can see that for average deaths, and recover cases, model’s simulated approximated observed average deaths and average recover cases. The average deaths and recovers cases for both variables lie within 95% prediction interval. Regarding daily new cases, the average number of observed cases are little outside of 95% prediction interval, however, the difference is not higher. The table show that the model predicted the observed data well. |
| 2 | The paper heavily focuses on the quantitative analysis only and does not analyze how and to what extent measures, including the closure of international borders, lockdowns, etc. helped to control infection trends in different countries? A short discussion could be insightful. | Actually, the basic purpose of this paper is forecasting daily deaths, new infections and recover cases using Bayesian Dynamic Linear Models (BDLMs). The title of the paper has been changed in the revised manuscript. However, a short discussion is added in the discussion section of the paper and given below:  “It can be observed that the results of this study are stable and are consistent with the trends of considered variables. The possible reasons may include: the techniques we used consider the dynamic nature of the system; secondly, more observations have been used in this study and perhaps this also exaggerated the findings. It is imperative to state that forecast is a complicated subject, therefore, these results could change due to various reasons including government policies regarding the current or future situation of COVID-19. This may include lockdown, closure of institutions or reducing the number of employees in a day at offices etc. The results of Meo at al. (2020) suggested that 15 days after the lockdown, daily new infections due to COVID-19 and growth factor of this disease showed decreasing trend, however, there was no significance decline in the mortality and prevalence in 27 randomly selected countries. Valero et al. (2020) investigated the impacts of lockdown and social distancing with deaths due to COVID-19 in 16 European countries. Their results suggested that there was close relationship between the deaths due to COVID-19 and the days elapsed until lockdown, however. However, there was week relationship between deaths and social distancing. There are other studies which investigated that screening, quarantine, isolation in different settings and contact tracing can help in reducing the new infections due to COVID-19 (Girum et al., 2020; Nouvellet et al., 2021).” |
| 3 | For some instances the predicted value is higher and for other instances, it is lower than the real values. One of the reasons for the ambiguity could be a change in control policy and action. The authors are suggested to explain the ambiguity. | Usually, it is hard to predict exact values by using model and particularly when a time series has high variability. A famous statistician Box G. P. E (1976) said that “all models are wrong, but some are useful”. If we look at all three figures about daily new infections, deaths and recover cases, it is obvious that the model closely predicted the observed data for daily new infections and deaths. However, for daily recover cases, the model underestimated some values and overestimated other values. In our opinion, the time series of daily recover cases has higher variability as compared to daily new infections and deaths. This may be one of the reasons that the predicted values are lower/higher than the real values for daily recover cases.  The control policy and action may not be the reason here as we already had the recorded time series data. The model will not have impacts of this, however, in case of high variability (higher maximum and minimum values), some values are underestimated and overestimated. |
|  | **Reviewer # 3** | |
| **Specific comments** | | |
| 1 | Previous related articles in the introduction could be mentioned in a more detailed critical way, that is stating their merits and drawbacks in a clear way. | The previous work related to the work of this paper has been mentioned in more details and critically with their merits and drawbacks in the introduction section of the revised manuscript. |
| 2 | The value of the forecasts cannot be judged without having other models as benchmarks. At the very least there should be some naive benchmarks. | The basic purpose of this research work is to model and forecast daily infections, deaths and recover cases due to COVID-19 in Pakistan. However, in the introduction section of the manuscript, a critical review is given where the merits and demerits of different models used for forecasting are mentioned. The performance of the model is assessed by comparing the distribution of observed and simulated data graphically as well as numerically. |
| 3 | The forecast precision is judged with graphical means without any numerical backing, for which plenty of error metrics exist. Plots do not reveal the detail about the forecast errors. | There are two approaches for evaluating the performance of the model. One is graphical and other is numerical.  Yes, the reviewer point is valid about the performance evaluation of the model by graphical means. Therefore, the models are evaluated numerically by comparing the distribution of the observed and model’s simulated data for daily new infections, deaths and recover cases in the revised manuscript. |
| 4 | For the benefit of the reader it would be good to be explicit about how forecasts are produced for different horizons (1, 2, ... , 20 days). | This is mentioned in detail in section 3.4 of the revised manuscript for one step ahead forecast. In addition, in the end of this section, k-step ahead forecast is given. Moreover, further references are added to assist the readers regarding forecasting with dynamic liner model. |
| 5 | 4. Lines 58-61: Add references to back the statements. Be explicit about what "mechanistic models" are. Do you mean state-space models? Do you mean compartmental epidemiological models? | Yes, "mechanistic models" means compartmental epidemiological models in our manuscript. A reference has been added in the revised manuscript. |
| 6 | 5. Lines 115 and 116: Name the variables in such a way that cases, deaths and recoveries are distinguished clearly from each other. Does COV_t represent all of them? It is also helpful to illustrate the dependencies of states. See for example <https://www.mdpi.com/2036-7449/13/1/27/htm> or <https://onlinelibrary.wiley.com/doi/epdf/10.1002/sim.2566> | Yes, COV represent all of them, which mean daily new infections, deaths and recoveries. Equations (1) and (2) can be write for each of these variables separately. For instance, equation (1) and (2) for daily new infections (DNI) can be write as follow:  ${DNI}_{t}= F_{t}\theta_{t}+ \epsilon_{t} \varepsilon_{t}\sim N(0, E_{t})$  $\theta_{t}= G_{t} \theta_{t-1}+ \omega_{t} \omega_{t}\sim N(0, W_{t})$  Where DNI stands for daily new infections. Similarly, we can express daily deaths and recover cases by using the above equations. It has been changed in the revised manuscript.  The paper mentioned have used dynamic models but they are different. For instance, in paper “<https://www.mdpi.com/2036-7449/13/1/27/htm>”, the authors used a Markov model which is different than our model. They collected data for 553 Polymerase Chain Reaction (PCR)-positive COVID-19 patients admitted to hospital whose eventual outcomes were known. The data included demographics, comorbidities and laboratory values taken at admission and throughout the course of hospitalization. Then they used multivariate Markov prognostic models to identify high-risk patients at admission along with a dynamic measure of risk incorporating time-dependent changes in patients’ laboratory values.  In the paper “<https://onlinelibrary.wiley.com/doi/epdf/10.1002/sim.2566>” the author used Bayesian network and transition probabilities.  In the above mentioned both papers, the authors used different models than we used. The dependency structure in our manuscript is about states of the system and observation. |
| 7 | 6. Equations 1 and 2: The details of F_t and G_t are not shown. What is their formulation for your model? | Equation (1) and (2) are given below for daily new Infections (DNI):  ${DNI}_{t}= F_{t}\theta_{t}+ \epsilon_{t} \varepsilon_{t}\sim N(0, E_{t})$  $\theta_{t}= G_{t} \theta_{t-1}+ \omega_{t} \omega_{t}\sim N(0, W_{t})$  Where $\boldsymbol{G}_{\boldsymbol{t}}$ linear system operator. In time series setting $\theta_{t}$ will have elements corresponding to various components of the time series process, like trend, seasonality, etc.  We observe a linear combination of the states with noise and matrix $\boldsymbol{F}_{\boldsymbol{t}}$  is the observation operator that transforms the model states into observations. Both observation and system equations can have additive Gaussian errors with covariance matrices $E_{t}$ and $W_{t}$.  In our case, we consider a polynomial model of order one, or random walk plus noise model, which is a univariate model with univariate state vector defined by:  ${DNI}_{t}= \theta_{t}+ \epsilon_{t}$  $\theta_{t}= \theta_{t-1}+ \omega_{t}$  For further details we refer to Petris (2010) and Johnson et al. (2018). |
| 8 | 7. Lines 140-141: The dependencies in your model should be made explicit. In them, the variables should be clearly included. COV seems to be a package or an aggregation of variables (infections, deaths, recoveries). Is formulation of dependencies in lines 140 and 141 generic, or the actual dependencies in your model? | The dependencies in Lines 140-141 is about the states and observation where $\theta_{t}$represent the state of the system at time t and ${COV}_{t}$ (${DNI}_{t}$ in the revised manuscript) represent the observation at time t. Details about states of the system has been mentioned in the methodology section of the manuscript.  Yes, COV is a package which represent daily new infections, deaths and recoveries. Equations (1) and (2) can be written for each of these variables separately. For instance, equation (1) and (2) for daily deaths can be write as follow:  ${DNI}_{t}= F_{t}\theta_{t}+ \epsilon_{t} \varepsilon_{t}\sim N(0, E_{t})$  $\theta_{t}= G_{t} \theta_{t-1}+ \omega_{t} \omega_{t}\sim N(0, W_{t})$  Where DNI stands for daily new infections. Similarly, we can express daily deaths and recover cases by using the above equations.  The dependence structure can then be written as below:  $\theta_{0}\to\theta_{1} \to\theta_{2}\to\ldots\to\theta_{t-1}\to\theta_{t}\to\theta_{t+1}\to\ldots$  $\begin{matrix} \downarrow\downarrow\\ {DNI}_{1} {DNI}_{2} \ldots\end{matrix}\begin{matrix} \downarrow\downarrow\downarrow\\ {DNI}_{t-1} {DNI}_{t} {DNI}_{t+1} \end{matrix}$  Similarly, we can write for daily deaths (DD) and daily recover cases (DRC) by replacing DNI by DD and DRC, respectively, in the above expressions. |
| 9 | 8. Section 3.3: It is not clear how the dependency structure of the model (which was not clearly added) was constructed or validated. An article that shows construction via cross-correlation and validation via examining posterior probabilities is the following:  <https://onlinelibrary.wiley.com/doi/epdf/10.1002/sim.2566> | Section 3.3 is about model’s evaluation. In this section, we evaluated out fitted model graphically as well as numerically. Graphically, we compared the probability density function of observed data with model’s simulated data. Also, the residuals of the fitted models were analyzed for checking model assumptions. Numerically, the fitted model was evaluated by comparing distribution of observed data with model’s simulated. |
| 10 | 9. Lines 165-167: The reasons for the choice should be stated. | The purpose here is to see whether the model reproduce the distribution of observed data or not. Here the distribution includes minimum value, first quartile, mean, median, third quartile and maximum value of the data. So, if the model’s simulated data reproduce closely the distribution of the observed data, then we can say that the performance of the model is good.  The wording in the revised manuscript has been change. |
| 11 | 10. Lines 301-302: Maximum and minimum figures do not correspond with the wording. You surely mean: minimum 2,132; maximum 4,149 | This has been corrected in the revised manuscript.  You, minimum number of new infections is 2,132; and maximum number of new infections is 4,149. It is now correctly mentioned in the text of the revised manuscript. |
| 12 | 11. About the data: WHO is cited (lines 54 and 55), but the citation directs to <https://www.worldometers.info/coronavirus/> There must be more clarity on which data is taken from which source. | The references have now been corrected in the revised manuscript. We have used data from both sources and therefore, both are cited and mentioned in the references list. |
|  | **General comments:** | |
|  | Overall, the writing of the article seems rushed. This is understandable in the current situation for many researchers. But for the benefit of the readers and the scientific community, it is important to clearly assert the decisions made in the research, supporting them with graphic means if feasible and adding enough detail of the models. To this purpose, the authors can look at previous articles of the same type. Careful writing can also improve the quality of the work. Expressions such as "[...] forecast is a tricky-subject [...]" should be articulated in a more helpful way.  The main criticism to the study is that, while it aims at forecasting scenarios, those are not included. Scenarios are different situations that can be modelled and assessed through different means. For example, by varying the underlying assumptions, or the initial conditions of the model; or by assuming different initial populations in some models; or by considering the presence or absence of government interventions. The way scenarios are formulated varies, partly depending on the type of model, and there is ample freedom to do this. In the article they are not explicitly included.  A sample study with scenarios determined by how authors modelled epidemic growth can be found at <https://www.mdpi.com/2077-0383/9/2/523>  This one defines scenarios based on the basic reproductive number, R0: <https://www.medrxiv.org/content/10.1101/2020.03.16.20036939v1.full.pdf>  This one explores scenarios based on changing the proportion of infected with respect to other groups of people: <https://journals.plos.org/plosone/article?id=10.1371/journal.pone.0230405>   The absence of scenarios, having aimed the article at it, is a major issue. On this ground, the article, in my view, should be rejected. One possibility for the authors could be to submit their work under a different title, not including scenarios. Even in that case, the other comments provided are applicable, which would lead to major changes, aimed at demonstrating that the research is of a high standard. The sample publications mentioned in the comments might be helpful. | The authors agree with the reviewer and grateful for pointing out this extremely important point. Therefore, the manuscript is revised with changed title from “FORECASTING FUTURE SCENARIOS OF COVID-19 BY USING BAYESIAN DYNAMIC LINEAR MODELS IN PAKISTAN”  to  “FORECASTING DAILY NEW INFECTIONS, DEATHS AND RECOVERY CASES DUE TO COVID-19 IN PAKISTAN BY USING BAYESIAN DYNAMIC LINEAR MODELS ”. |

**References**

Castillo O, Melin P, (2020) Forecasting of COVID-19 time series for countries in the world based on a hybrid approach combining the fractal dimension and fuzzy logic. Chaos, Solitons and Fractals 140 (2020) 110242. DOI: <https://doi.org/10.1016/j.chaos.2020.110242>.

Meiln P, Monica JC, Sanchez D, Castillo O, (2020) Multiple Ensemble Neural Network Models with Fuzzy Response Aggregation for Predicting COVID-19 Time Series: The Case of Mexico. Healthcare 2020, 8, 181; doi:10.3390/healthcare8020181.

Boccaletti S, Atangana A, (2020) Modeling and forecasting of epidemic spreading: The case of Covid-19 and beyond. Editorial / Chaos, Solitons and Fractals 135 (2020) 109794. DOI: <https://doi.org/10.1016/j.chaos.2020.109794>.

Sun T, Wang Y, (2020) Modeling COVID-19 epidemic in Heilongjiang province, China. Chaos, Solitons and Fractals 138 (2020) 109949. DOI: <https://doi.org/10.1016/j.chaos.2020.109949>.

 Box GEP, (1976) [Science and statistics](http://www-sop.inria.fr/members/Ian.Jermyn/philosophy/writings/Boxonmaths.pdf). [*Journal of the American Statistical Association*](https://en.wikipedia.org/wiki/Journal_of_the_American_Statistical_Association), **71** (356): 791–799, DOI: [10.1080/01621459.1976.10480949](https://doi.org/10.1080%2F01621459.1976.10480949).

Meo SA, Abukhalaf AA, Alomar AA, AlMutairi FJ, Adnan Mahmood Usmani AM, Klonoff DC (2020) Impact of lockdown on COVID‑19 prevalence and mortality during 2020 pandemic: observational analysis of 27 countries. Eur. *J. Med. Res. 25:56.* DOI: <https://doi.org/10.1186/s40001-020-00456-9>.

Valero CM, Miranda JD, Sánchez FJM, (2020) The Impact of time to impose lockdown on COVID-19 cases and deaths in European countries. *Scientific letter/Med Clin (Barc),* [Volume 155, Issue 10](https://www.sciencedirect.com/science/journal/23870206/155/10), Pages 459-460. DOI: <https://doi.org/10.1016/j.medcle.2020.07.011>.

Girum T, Lentiro K, Geremew M, Migora B, Shewamare S, (2020) Global strategies and effectiveness for COVID-19 prevention through contact tracing, screening, quarantine, and isolation: a systematic review. Tropical Medicine and Health (2020) 48:91 DOI: <https://doi.org/10.1186/s41182-020-00285-w>.

Nouvellet B, et al., (2021) Reduction in mobility and COVID-19 transmission. NATURE COMMUNICATIONS, 12:1090. DOI: <https://doi.org/10.1038/s41467-021-21358-2>.

Petris G (2010) An R Package for Dynamic Linear Models. *Journal of Statistical Software*, Volume 36, Issue 12.

Johnson M, Caragea PC, Meiring W, Jeganathan C, Atkinson PM (2018) Bayesian Dynamic Linear Models for Estimation of Phenological Events from Remote Sensing Data. Journal of Agricultural, Biological, and Environmental Statistics, Volume 24, Number 1, Pages 1–25. DOI: <https://doi.org/10.1007/s13253-018-00338-y>.
